# Facial Skin Revitalization With CPM‐HA20G (Hyaluronic Acid + Glycerol): A Comparative Case Series Using Three Delivery Techniques With Ultrasound Confirmation

**DOI:** 10.1111/jocd.70660

**Published:** 2026-01-11

**Authors:** Kim Booysen, Frank Lin

**Affiliations:** ^1^ Private Practice Private Practice London England UK; ^2^ Plastic Surgery Eastern Plastic Surgery Melbourne Victoria Australia

## Abstract

**Background:**

Skin boosters are commonly administered into the dermis or immediate subdermal plane using serial puncture technique. Alternative delivery methods, such as blunt cannulas and multiple‐needle injector devices, are available, but their ability to achieve comparable clinical outcomes has been under‐investigated. This case series presents the first prospective comparative evaluation of CPM‐HA20G administered via three different modalities, assessing efficacy, safety, and adverse events, while utilizing ultrasound to confirm product placement. To minimize patient‐related variability, a split‐face study design was also employed.

**Objective:**

To demonstrate the efficacy, safety, and comparability of results when delivering CPM‐HA20G via multiple modalities into the immediate subdermal plane.

**Methods:**

Fifteen patients received three treatments of CPM‐HA20G, four weeks apart, using serial puncture, blunt cannula, and multiple‐needle injector device. A separate split‐face case compared serial puncture with blunt cannula delivery in the same patient, with ultrasound confirmation of product placement. Satisfaction rates, pain scores, and skin quality were assessed at 4, 8, and 12 weeks.

**Results:**

All 15 patients across the three modalities achieved comparable improvements in skin elasticity, firmness, and hydration. High patient satisfaction was reported, with no serious adverse effects observed. A paired *t*‐test showed pain scores were significantly lower with blunt cannula (mean 2.2) compared with serial puncture (mean 4.6) and injector device (mean 4.5) (***p* < 0.0001**). Blunt cannula delivery was also associated with a lower incidence of bruising, suggesting clinical advantages for patient comfort and recovery.

**Conclusion:**

This case series demonstrates that serial puncture technique, blunt cannula and multiple‐needle injector device can be used to deliver safe, comparable, and effective CPM‐HA20G treatments. These findings highlight comparable efficacy across modalities and suggest blunt‐cannula delivery enhances patient comfort and recovery time.

## 1 Introduction

Skin boosters are typically administered to the dermis or immediate subdermal plane using the serial puncture technique. Although alternative delivery modalities such as blunt cannula and multiple‐needle injector devices are available, their ability to achieve comparable outcomes has not been comprehensively investigated. Previous studies on skin boosters have largely focused on serial puncture delivery, with only limited data describing cannula use and almost none evaluating injector devices. Moreover, many prior studies did not utilize ultrasound to confirm placement of the product within the intended plane.

Current manufacturer injection guidelines advocate that CPM‐HA20G should be injected in the mid to deep dermis [1]. However, a consensus paper on the use of hyaluronic acid fillers from the cohesive polydensified matrix (CPM) range also indicated that CPM‐HA20G can be injected in the dermal or immediate subdermal plane [2]. Studies of the physicochemical properties of CPM‐HA20G, including extrusion force, swelling degree, rheological performance, and cohesivity, indicate that it is well suited to superficial injection when compared with other similar skin‐quality boosters [3].

The BELOVE study showed that treatment in the subdermal plane revitalizes the skin with improvements in skin elasticity, firmness, tone, radiance, hydration, and a reduction in roughness. In this study, treatments consisting of 50 μL were distributed over the lower cheeks of 25 healthy female subjects with signs of facial skin aging, using approximately 20 serial injection points at an immediate subdermal level with a 30G ½ needle. Three treatments were performed one month apart. Objective physiological investigations showed improvements in skin tone, roughness, hydration and radiance, that were observed up to 36 weeks [4].

Previous skin‐quality booster studies have focused on serial puncture techniques as the primary method for treatment delivery [3, 4], but treatment with a blunt cannula has also been described [5, 6]. However, delivery of CPM‐HA20G via blunt cannula or multiple‐needle injector device has not been assessed before. Many of the initial studies on skin revitalization also did not utilize ultrasound to confirm placement of the product in the dermis or subdermal plane.

**FIGURE 1:**
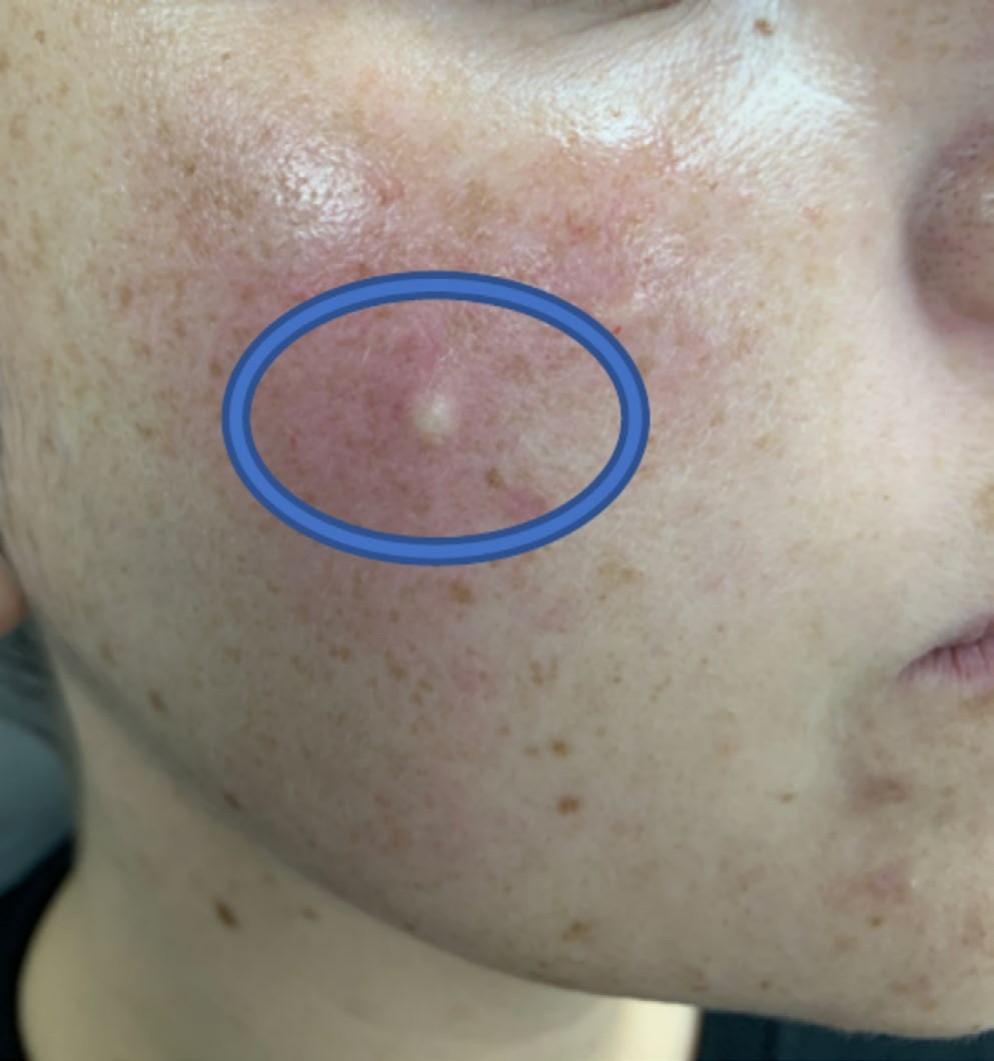
Subject's superficial HA bleb following intradermal injection of CPM‐HA20G.

**FIGURE 2:**
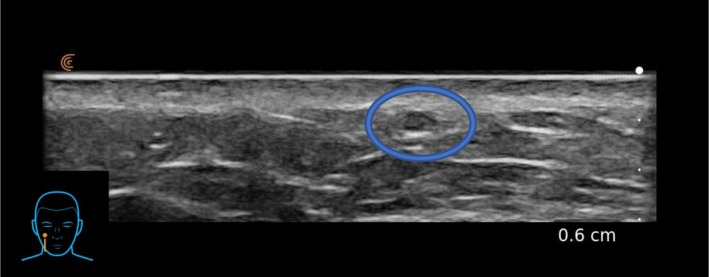
Subject's superficial HA bleb as seen on ultrasound imeediately after intradermal injection of CPM‐HA20G.

The authors are experienced users of ultrasound for hyaluronic acid (HA) assessment and use this modality in clinic to review placement of HA within the tissues. HA is a hydrophilic gel, that reflects ultrasonic sound waves to a lesser degree and appears as an anechoic or hypoechoic lesion in the tissues after injection. This allows injectors with ultrasound experience to confirm the correct placement of HA post‐injection [7]. The authors both used the Clarius L20, a 20 MHz, linear probe handheld ultrasound for HA assessment. The authors each investigated the depth of CPM‐HA20G placement after attempting to treat the dermal plane with needle gauges of 30G to 34G. CPM‐HA20G was consistently observed in the immediate subdermal plane on ultrasound, immediately after injection, despite an intradermal appearance on visual inspection (Figures 1 and 2). The cause for this could be a result of injection technique, HA particle behavior, the anatomical plane of injection, individual tissue quality, and tissue response to filler placement [8, 9, 10, 11].

**FIGURE 3:**
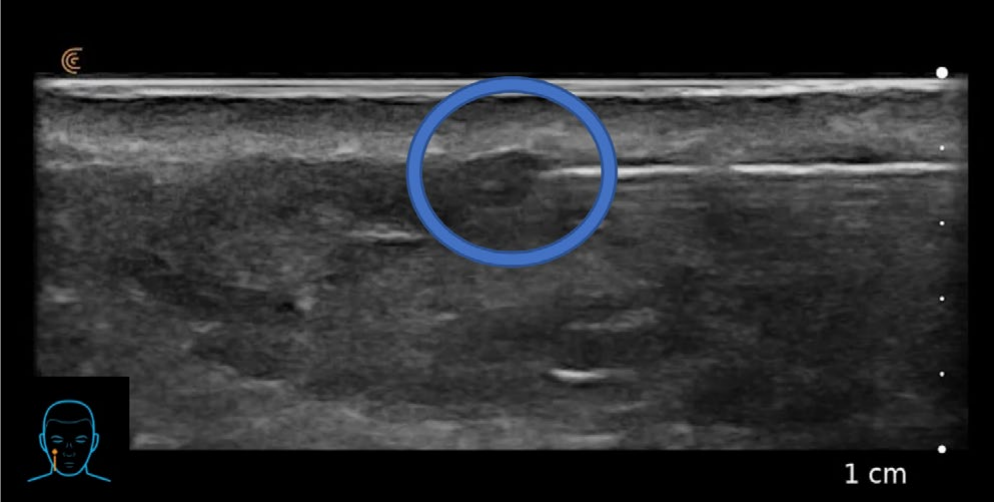
Subject's superficial HA placement on ultrasound during subdermal linear‐thread injection of CPM‐HA20G using a 25G 38 mm cannula.

Further ultrasound investigation showed that CPM‐HA20G could also be placed in the same immediate sub‐dermal plane using a 25G cannula (Figure 3).

A multiple‐needle injector can also be used to accurately place CPM‐HA20G in the dermal or immediate subdermal plane. Ultrasound assessment confirmed dermal thickness and, therefore, the correct depth of treatment. However, because the volume delivered at each injection site was very small, the product could not be convincingly visualized on ultrasound after treatment.

The authors thus proposed that a treatment with CPM‐HA20G could be performed using a blunt cannula or multiple‐needle injection, without compromising treatment outcomes and potentially lowering adverse events (AEs) such as pain and bruising. To the authors' knowledge, no prior study has directly compared CPM‐HA20G delivery by needle, cannula, and injector using ultrasound to confirm placement. This study aimed to address that gap.

## 2 Methods

**FIGURE 4:**
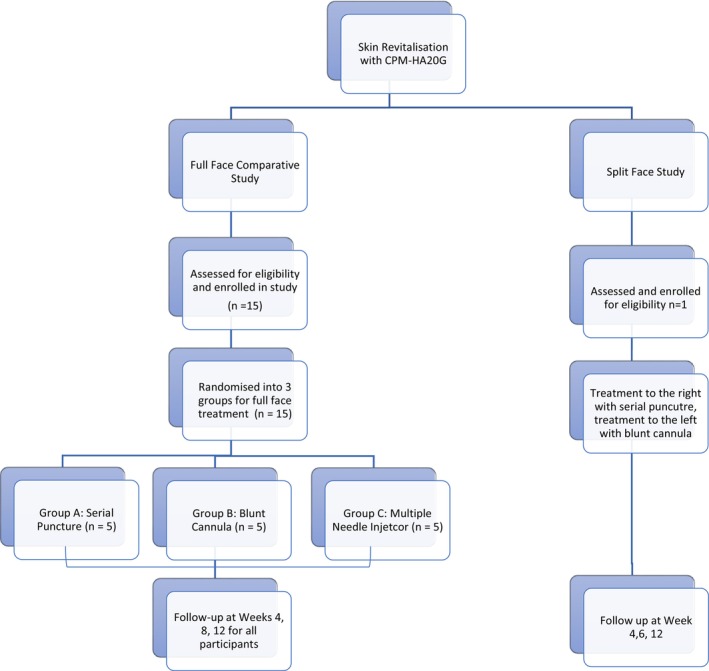
CONSORT flow diagram illustrating participant enrolment, allocation, follow‐up, and analysis across the two study arms, reported in accordance with the CONSORT 2010 guidelines [12].

The study was undertaken with two separate arms (Figure 4). One was a comparative study of 15 patients, where the serial puncture, blunt cannula, or multiple‐needle injector was used for three treatments of the entire face. A further individual split‐face study was performed, where three treatments were performed on opposing sides of the same patient's face using both the serial puncture and blunt cannula technique. Written consent was obtained from all participants and the study was conducted in accordance with the principles of the 1975 Declaration of Helsinki.

### 2.1 Full Face Comparative Study

Fifteen patients, fourteen female and one male, with a mean age of 38.5 (range 28–52), were treated with CPM‐HA20G to each side of their mid and lower face for skin‐quality improvement. Patients were Caucasian, East Asian, or Southeast Asian in background with Fitzpatrick score range between 2 to 5. Patients were randomly assigned to treatment groups based on their order of enrolment, using an automated randomization table.

No formal sample‐size calculation was performed. This was an exploratory, descriptive case series intended to generate preliminary comparative data across delivery techniques.

Patients were screened for any contraindications to hyaluronic acid treatment and were excluded if they had previous HA, polynucleotide, or other skin‐booster treatments in the preceding 12 months.

**FIGURE 5:**
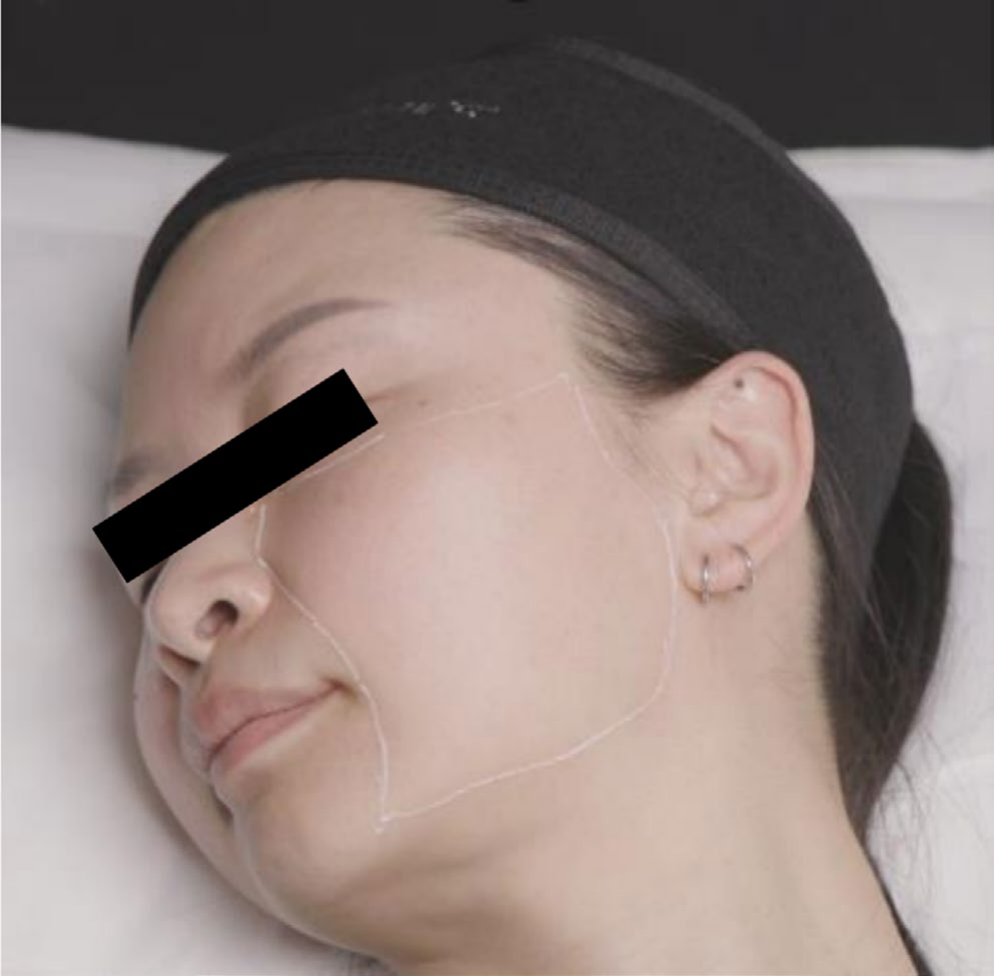
The treatment zone.

The treatment zone is superiorly bounded by the body of the zygoma and inferior orbital margin; medially by the nasal sidewall, nasolabial fold, and marionette line; inferiorly by the mandibular margin; and posteriorly by the preauricular sulcus, as shown in Figure 5.

About 1 mL of CPM‐HA20G was administered to each side of the face per session, with a total of three sessions delivered at four‐week intervals. Five patients received treatment using the serial puncture technique, five received treatment using a cannula, and five with a multiple‐needle injector device, specifically the Vital Injector 2 from Eunsung Global Corp., based in Gangwon‐do, Korea.

Patients were reviewed at initial assessment and at 4, 8, and 12 weeks after their first treatment session. Following each treatment session, patients were assessed for their treatment pain score and evaluated by the treating clinician for adverse events. At each of the follow‐up time points, patients completed a GAIS questionnaire assessing their satisfaction relating to skin hydration, skin tone evenness, skin surface evenness, skin elasticity, and adverse events.

Our primary end points were the comparison of pain score and patient satisfaction between the three injection methods. Secondary endpoint was the assessment of safety based on clinician assessment and patient‐reported AEs.

Quantitative variables were described using the mean, SD, and range. Pain score and patient satisfaction were assessed using the paired *t*‐test. Changes were considered significant at *p* < 0.05. Statistical analysis was performed using Microsoft Excel For Mac V16.54.

In this exploratory case series, clinical significance was defined as an improvement that was perceptible and meaningful to the patient or clinician, rather than solely based on statistical testing. For the pain score (0–10 numeric scale), a reduction of ≥ 2 points was considered clinically meaningful. For GAIS (0–3 scale), an improvement of ≥ 1 category (e.g., from ‘no change’ to ‘improved’ or higher) was considered clinically significant, as it reflects a visible and patient‐perceived enhancement in skin quality.

### 2.2 Serial Puncture Method

**FIGURE 6:**
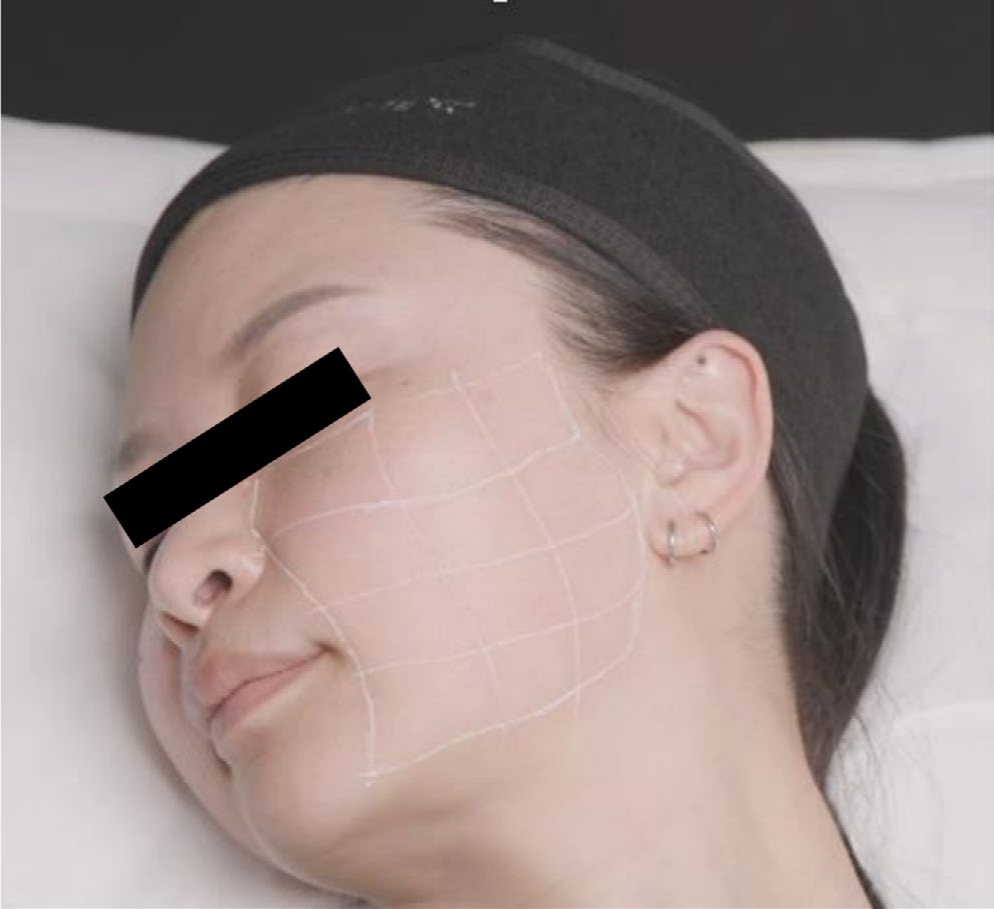
Treatment zone divided into 12 rectangles for serial puncture treatment.

The treatment zone of each side of the face was divided into 12 rectangles (Figure 6). Topical anesthesia (10.56% lidocaine gel) was used and left on the skin for 30 min prior to treatment. A 30G, 0.3 x 13 mm needle was used to deliver four injections of approximately 0.02 mL CPM‐HA20G to each square in the intradermal or immediate subdermal plane. This resulted in 48 injections and total volume of 1 mL delivered to each side of the face.

### 2.3 Blunt Cannula Method

**FIGURE 7:**
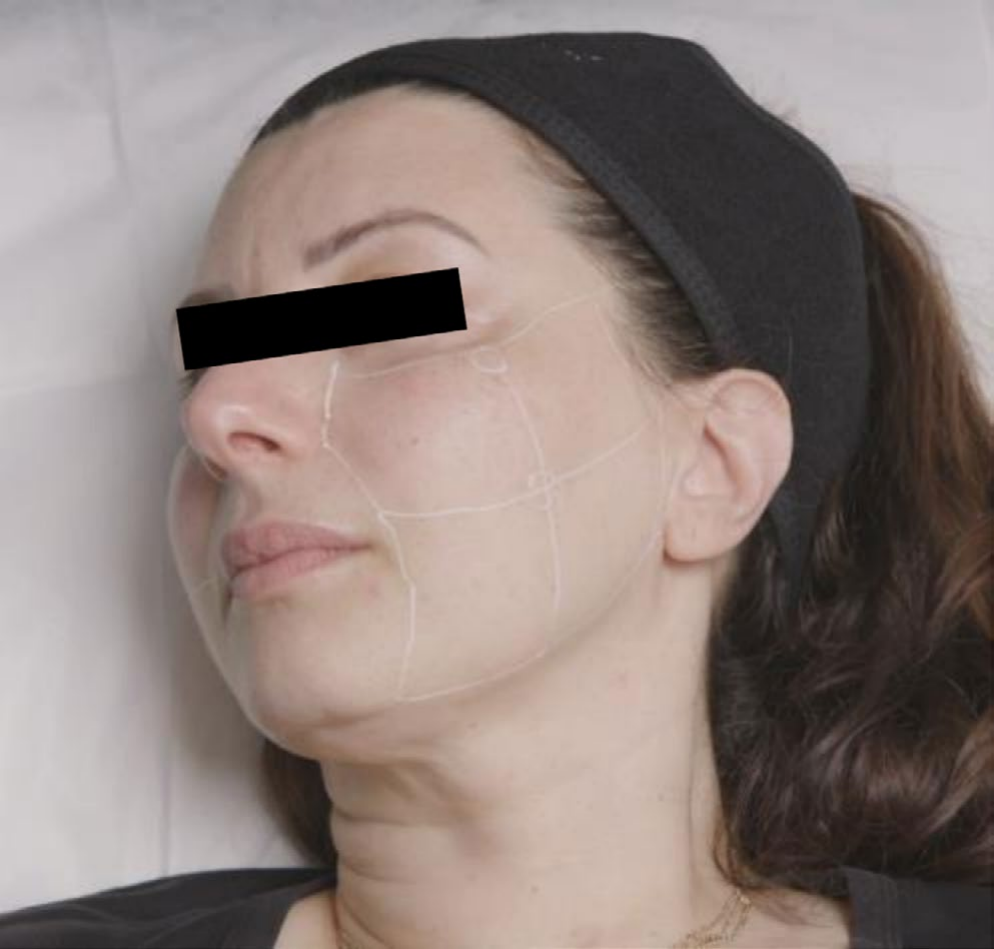
Treatment zone divided into four equal rectangles for blunt cannula treatment.

The treatment zone was divided into four equal rectangles (Figure 7). The central point of the treatment zone and the midpoint of the upper border were marked and infiltrated with 0.1 mL of lignocaine 1% with adrenaline (1:100000). A 25G, 5‐cm blunt cannula was used to place twelve 0.02 mL threads of CPM‐HA20G into each of the quadrants, in the immediate subdermal plane. This resulted in a total volume of 1 mL delivered to each side of the face.

### 2.4 Multiple‐Needle Injector (Vital Injector 2) Method

Patients were prepared with topical anesthesia (10.56% lignocaine gel) applied for 30 min prior to treatment. The multiple‐needle injector was loaded with a 9‐pin injection hub, with the injection depth set to 1 mm. About 1 mL of CPM‐HA20G was diluted with the addition of 2 mL of sterile normal saline, making a total volume of 3 mL administered to each side of the face. The mixture was delivered through an average of 50 injections to each side of the face using the multiple‐needle injector.

The placement of the product in the subdermal plane had been confirmed with ultrasound on several previous occasions and therefore not repeated for each individual case using the cannula and serial puncture technique. The delivery of product using the multiple‐needle injector is depth specific, but product amounts are too small to be convincingly confirmed with ultrasound investigation.

### 2.5 Split‐Face Study

A 54‐year‐old female patient with no relevant medical history and no previous HA, polynucleotide, or other skin‐booster treatments in the preceding 12 months was treated with two different modalities. The primary endpoints were the comparison of pain scores and patient satisfaction between the two injection methods. The secondary endpoint was the assessment of safety based on clinician evaluation and patient‐reported AEs.

The patient was prepared with topical anesthesia (LMX 4%) applied for 30 min prior to treatment. The patient underwent three 1 mL CPM‐HA20G treatments to each side of the face. The right side of the face was treated using a serial puncture technique and the left with a blunt cannula technique.

On the right side of the face, a 30G, 0.3 × 13 mm needle was used to deliver 0.02 mL/injection point with approximately 48 injection points from the malar mound to the angle of the mandible. A grid of 12 equal squares as shown in Figure 5, was used to aid even distribution of the product, with four injection points of 0.02 mL placed per square. The placement in the subdermal plane was confirmed with ultrasound at each treatment.

On the left‐hand side of the face, the treatment zone was similarly divided into four equal rectangles as shown in Figure 6. The central point of treatment zone and the midpoint of the upper border were marked, and infiltrated with 0.2 mL of lignocaine 2%. A 25G, 38 mm blunt cannula was used to place twelve 0.02–0.05 mL threads of CPM‐HA20G to each of the quadrants, in the immediate subdermal plane. This resulted in a total volume of 1 mL delivered to each side of the face. The placement in the subdermal plane was confirmed with ultrasound at each treatment. The treatment was repeated at monthly intervals, for a total of three treatments.

Patient and injector satisfaction were assessed using the GAIS score at each follow‐up session, with photographic assessment prior to treatment and one month after each of the three treatments. The patient was also asked to rate the changes in skin smoothness, tone, hydration, and elasticity using the GAIS score. The patient was asked to rate their pain score during treatment on a numeric rating scale of 0–10, with 0 being no pain and 10 being the worst pain ever experienced. The patient was also asked to report any AEs during and after treatment.

The split‐face case was included as a feasibility sub‐study to directly compare serial puncture and blunt cannula delivery within the same patient, thereby eliminating inter‐individual variability in factors such as skin type, age, and prior treatment history. This design allowed within‐subject comparison of pain, placement accuracy (confirmed by ultrasound), and clinical outcomes, serving to validate the comparability of the two modalities under identical patient conditions. As this was a preliminary proof‐of‐concept assessment, only one subject was included.

## 3 Results

In the full‐face comparative study, the treatment population consisted of 14 female and 1 male patient. The average age was 38.5 years (range 28–52). The mean age for Group 1 was 36.6 years (range 26–49), Group 2 was 38.6 years (range 28–52), and Group 3 was 40.2 years (range 29–48). There were no significant differences in the demographics of the three treatment groups in terms of age or background.

Pain‐score results are presented in Table 1. Pain was scored out of 10, with 0 being no pain and 10 being worst pain. The overall mean pain score for the serial puncture group and the mutiple needle Injector group over three sessions were similar, at 4.6 and 4.5, respectively. There was a statistically significant reduction in the pain score for the blunt cannula group compared with both other groups with an overall mean pain score of 2.2 (*p* < 0.0001). The *p*‐value is presented only for the pain scores, as this is the sole parameter demonstrating a statistically significant difference between groups. All other outcomes were comparable, with no statistically significant differences, which supports the overall interpretation presented.

**TABLE 1:** Mean pain score for Groups 1–3.

	Session 1 (Week 4) Mean ± SD	Session 2 (Week 8) Mean ± SD	Session 3 (Week 12) Mean ± SD	Overall Session mean
Group 1: Serial puncture	4.8 ± 1.9	4.6 ± 0.9	4.4 ± 0.5	4.6
Group 2: Blunt cannula	2.6 ± 1.5	2.0 ± 0.7	2.0 ± 0.7	2.2
Group 3: Multiple‐needle injector	4.6 ± 0.8	4.4 ± 0.5	4.4 ± 0.5	4.5

Results from patient satisfaction questionnaire are presented in Table 2. Patient self‐reported assessment based on GAIS were converted to a numerical value, with “No change” designated as 0; improved designated as 1; much improved designated as 2; and very much improved designated as 3. No patients at any stage reported their results were in the worse, much worse, or very much worse category.

**TABLE 2:** Mean ± standard deviation GAIS score for Groups 1–3 based on patient reported satisfaction questionnaire at the week 4, 8, and 12 clinical reviews.

Questions ratings based on	Hydration	Skin tone evenness	Skin surface evenness	Elasticity*
	Have you noticed less skin dryness/better skin hydration post treatment?	Did you notice an improvement in your skin tone after the first treatment?	Did you notice an increase in smoothness and softness of the skin?	At this timepoint, how do you rate the elasticity in your skin compared to baseline?
Group 1: Serial puncture	Mean ± SD	Mean ± SD	Mean ± SD	Mean ± SD
Week 4	1.4 ± 0.9	1.0 ± 0.7	0.8 ± 0.4	0.6 ± 0.5
Week 8	1.6 ± 0.9	1.2 ± 0.8	1.2 ± 0.4	1.2 ± 0.4
Week 12	2.2 ± 0.8	1.6 ± 0.9	1.6 ± 0.5	1.4 ± 0.5
Group 2: Blunt cannula				
Week 4	1.2 ± 0.4	1.0 ± 0.7	1.2 ± 0.4	1.2 ± 0.4
Week 8	1.6 ± 0.5	1.4 ± 0.9	1.4 ± 0.9	1.4 ± 0.5
Week 12	2.2 ± 0.4	2.2 ± 0.4	2.2 ± 0.4	1.3 ± 0.6
Group 3: Multiple‐needle injector				
Week 4	1.6 ± 0.9	1.0 ± 0.7	1.4 ± 0.5	0.8 ± 0.4
Week 8	1.6 ± 0.4	1.4 ± 0.5	1.6 ± 0.5	1.2 ± 0.4
Week 12	2.4 ± 0.5	1.6 ± 0.5	2.0 ± 0.7	1.8 ± 0.4

* Elasticity defined as the skin's ability to stretch and bounce back.

**FIGURE 8:**
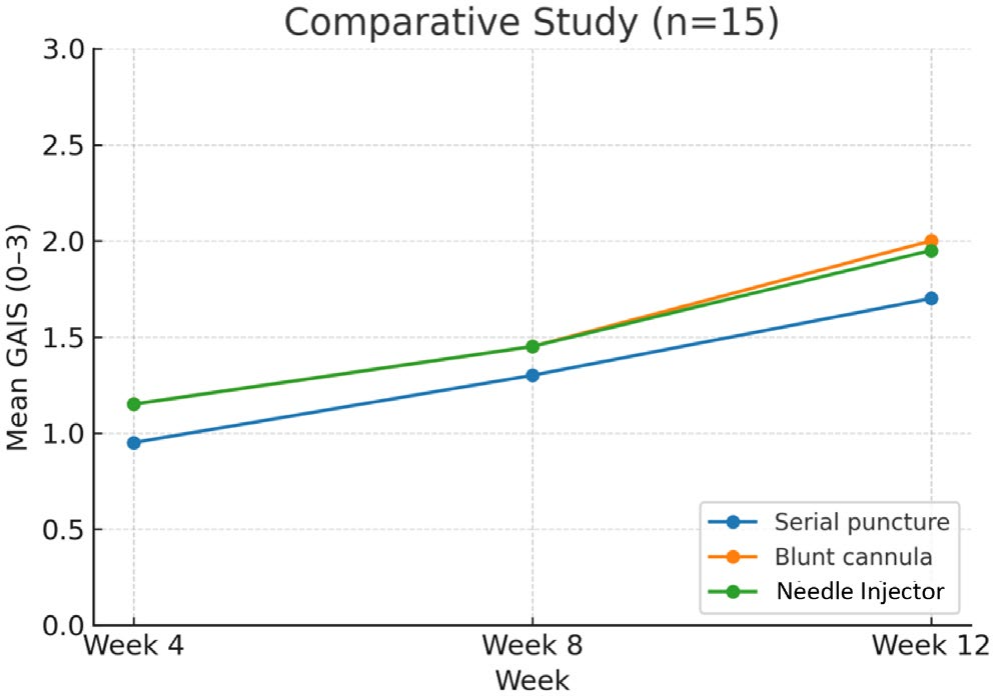
Change in mean GAIS (0–3 scale) from Week 4 to Week 12 by delivery technique in the comparative study. Values represent the average of hydration, tone evenness, surface evenness, and elasticity scores at each timepoint. Error bars not shown due to unavailability of dispersion metrics. Abbreviations: GAIS, Global Aesthetic Improvement Scale.

Results demonstrated no significant difference between the three groups. Notably, patient satisfaction improved from week 4 to week 12, after initial treatment in all categories irrespective of the delivery method (Figure 8 and Table 3).

**TABLE 3:** Mean GAIS Scores Over Time by Delivery Technique in the Comparative Study.

Timepoint	Serial puncture	Blunt cannula	Multiple‐needle injector
Week 4	0.95	1.15	1.15
Week 8	1.30	1.45	1.45
Week 12	1.70	2.00	1.95

AEs were assessed by the treating clinician immediately after each session and by patients at the Weeks 4, 8, and 12 using a questionnaire. No significant AEs were noted by the clinician in any categories.

Finally, patient satisfaction was assessed using a numeric scale [1, 2, 3, 4, 5, 6, 7, 8, 9, 10] and results are presented in Table 4. There was no statistically significant difference in the overall patient satisfaction between the three groups. Notably, satisfaction increased in all the three groups between week 4 to week 12.

**TABLE 4:** Mean ± standard deviation for overall patient satisfaction with treatment.

	Mean overall satisfaction with treatment 1 = Not satisfied at all 10 = Very satisfied
Group 1: Serial puncture	Mean ± SD
Week 4	7.2 ± 1.6
Week 8	7.4 ± 1.5
Week 12	8.4 ± 1.1
Group 2: Blunt cannula	
Week 4	7.6 ± 1.9
Week 8	8.25 ± 1.3
Week 12	9.0 ± 1.2
Group 3: Multiple‐needle injector	
Week 4	7.0 ± 1.0
Week 8	7.6 ± 0.9
Week 12	8.2 ± 0.4

In the split‐face study, the pain scores for each side of the face after treatment are shown in Table 5. Pain was scored out of 10, with 0 being no pain and 10 being worst pain. The average for the right (serial puncture technique) was 3.3 and for the left (blunt cannula technique) was 2.3.

**TABLE 5:** Pain scores for each side of the face after treatment with serial puncture on the right and blunt cannula on the left.

Split‐face study pain score	4 weeks		8 weeks		12 weeks	
	Right	Left	Right	Left	Right	Left
	4.0	3.0	3.0	2.0	3.0	2.0

The split‐face study patient's skin quality GAIS scores after the 1st and 2nd treatment were 1 “Improved”, but changed to 2 “Much Improved” after the 3rd treatment. Investigator scores improved similarly from 1 to 2 after the 2nd treatment.

There was no significant difference observed by the patient or injector, when comparing the right with the left side of treatment (Table 6).

**TABLE 6:** GAIS scores for each skin quality in the split‐face study.

	Hydration		Skin tone evenness		Skin surface evenness		Elasticity*	
Questions ratings based on	Have you noticed less skin dryness/better skin hydration post treatment?		Did you notice an improvement in your skin tone after the first treatment?		Did you notice an increase in smoothness and softness of the skin? Please give a rating.		At this timepoint, how do you rate the elasticity in your skin compared to baseline?	
Split‐face study	R	L	R	L	R	L	R	L
Week 4	1.0	1.0	1.0	1.0	1.0	1.0	1.0	1.0
Week 8	2.0	2.0	1.0	1.0	1.0	2.0	1.0	1.0
Week 12	2.0	2.0	2.0	2.0	2.0	2.0	2.0	2.0

* Elasticity defined as the skin's ability to stretch and bounce back.

The patient's overall GAIS scores after the 1st and 2nd treatment were 2 “Improved”, but changed to 1 “Much Improved” after the 3rd treatment. Investigator scores improved similarly from 1 to 2 after the 2nd treatment (Table 7).

**TABLE 7:** Overall treatment satisfaction in the split‐face study.

Split‐face study	Overall satisfaction with treatment. “No change” designated as 0; Improved designated 1; Much improved designated 2; and Very much improved designated 3.	
	Right (Serial puncture)	Left (Blunt cannula)
Week 4	1.0	1.0
Week 8	2.0	2.0
Week 12	2.0	2.0

**FIGURE 9:**
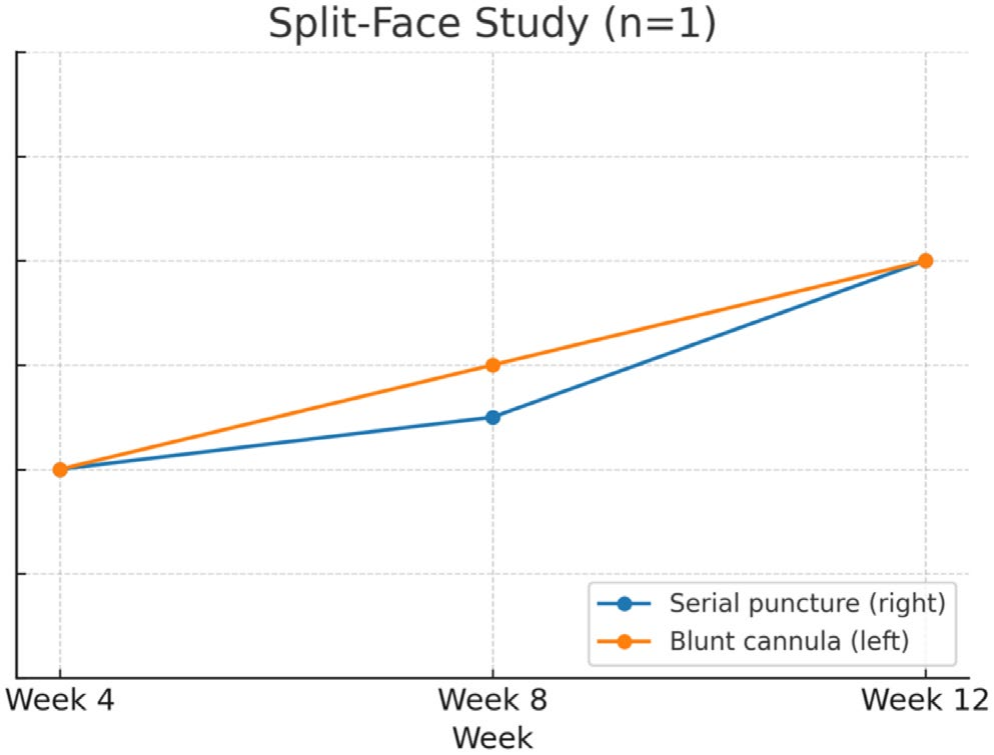
Change in mean GAIS (0–3 scale) over time in the split‐face study (*n* = 1) comparing serial puncture (right side) with blunt cannula (left side). Values represent means across four GAIS domains (hydration, tone evenness, surface evenness, and elasticity).

The mean GAIS scores in the split‐face study improved with each successive week (Table 8 and Figure 9).

**TABLE 8:** Mean GAIS scores over time by delivery technique in the split‐face study.

Timepoint	Serial puncture (Right)	Blunt cannula (Left)
Week 4	1.0	1.0
Week 8	1.3	1.5
Week 12	2.0	2.0

As this was a single‐patient feasibility comparison, the split‐face study was anecdotal in nature and intended to provide qualitative and procedural insights rather than statistical inference. The data were therefore not included in formal statistical testing and no power calculation was applicable.

AEs of small bruises that lasted less than 5 days were reported after each of the serial puncture treatments on the right side of the split‐face study. One bruise was reported on the left side after the 2nd treatment in the split‐face study. These all resolved spontaneously without intervention within 5 days (Table 9).

**TABLE 9:** Summary of adverse events across treatment groups.

Technique	Adverse event observed	Frequency/severity	Resolution
Serial puncture	Bruising	Multiple sessions; mild, transient	Spontaneous resolution < 5 days
Blunt cannula	Bruising	Single instance in split‐face patient; mild	Spontaneous resolution < 5 days
Multiple‐needle injector	None	—	—
Overall	No serious adverse events	Only mild, self‐limiting events recorded	All resolved without intervention

**FIGURE 10:**
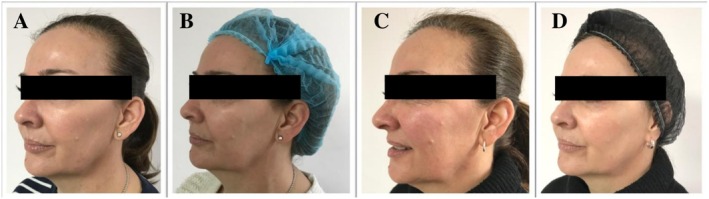
(A) Before treatment, (B) 4 weeks after 1st treatment, (C) 8 weeks after 2nd treatment, and (D) 12 weeks after 3rd treatment of the split‐face study.

**FIGURE 11:**
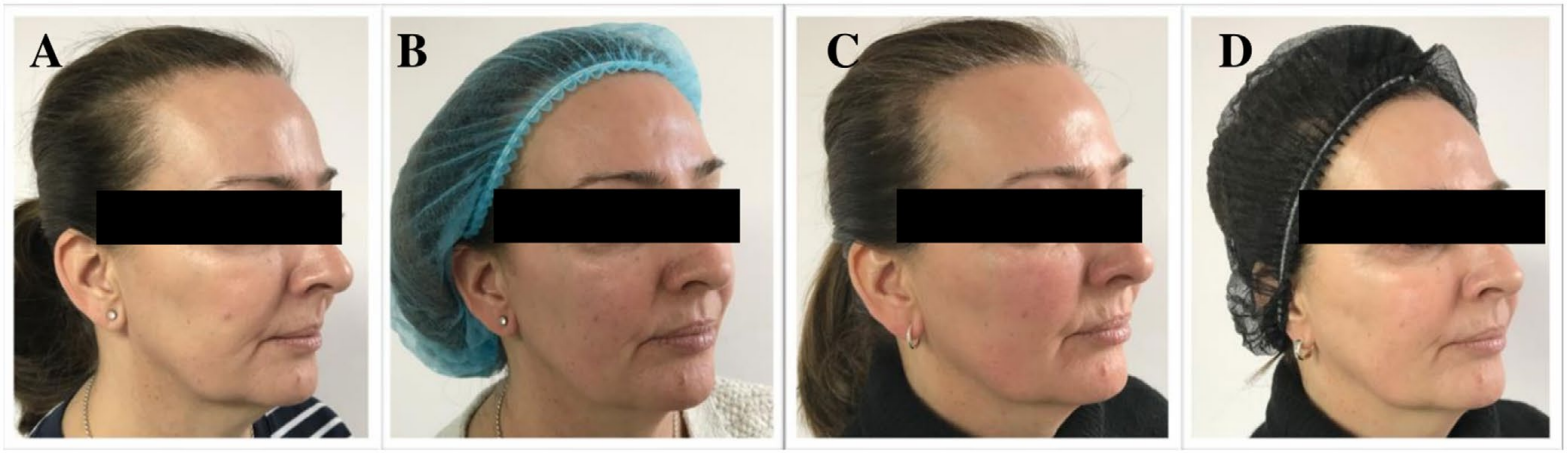
(A) Before treatment, (B) 4 weeks after 1st treatment, (C) 8 weeks after 2nd treatment, and (D) 12 weeks after 3rd treatment of the split‐face study.

**FIGURE 12:**
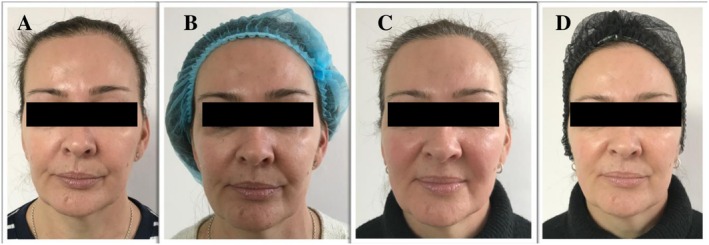
(A) Before treatment, (B) 4 weeks after 1st treatment, (C) 8 weeks after 2nd treatment, and (D) 12 weeks after 3rd treatment of the split‐face study.

Photographic assessment was performed prior to the split‐face treatment (Figures 10A, 11A and 12A) and at week 4 (Figures 10B, 11B and 12B), 8 weeks (Figures 10C, 11C and 12C) and 12 weeks (Figures 10D, 11D and 12D).

## 4 Discussion

This comparative case series demonstrates that CPM‐HA20G can be effectively delivered using serial puncture, blunt cannula, or multiple‐needle injector, with all three modalities producing consistent improvements in hydration, tone, surface evenness, and elasticity by 12 weeks. Importantly, ultrasound confirmed that CPM‐HA20G reliably deposited in the immediate subdermal plane when administered via cannula and serial puncture, reinforcing that the intended anatomical target can be reached across techniques. To our knowledge, this is the first study to compare CPM‐HA20G delivered by three techniques while using ultrasound to validate injection depth, providing new insights into how modality selection influences placement, comfort, and clinical outcomes.

The findings are directionally consistent with the BELOVE study, which demonstrated improvements in skin hydration, firmness, and radiance following serial puncture delivery into the subdermal plane [4]. Similarly, prior research comparing needles and cannulas has documented reduced bruising, lower pain scores, and improved patient tolerability with cannula injections, including the work of Spada et al. in tear trough treatments [5, 6]. Our results align with this evidence, showing that blunt cannula delivery offers a measurable comfort advantage without compromising efficacy.

The present study also extends current knowledge by demonstrating that a multiple‐needle injector—despite delivering micro‐aliquots too small to be visualized on ultrasound—can still achieve comparable clinical results. This suggests that consistent subdermal delivery, rather than modality itself, is the most important factor in determining treatment success with CPM‐HA20G. The convergence of domain‐specific GAIS improvements across all groups further reinforces the robustness of the product's effect irrespective of delivery method.

### 4.1 Clinical Implications

The pain reduction observed with blunt‐cannula injections has important implications for patient experience, particularly for individuals desiring minimal discomfort or with low pain thresholds. Reduced bruising with the cannula further supports its use in patients requiring minimal downtime or with social/work constraints. In contrast, serial puncture—while widely used—was associated with higher pain and more frequent bruising, consistent with previously published data.

From a practical perspective, cannula delivery may also help mitigate issues inherent to serial‐puncture techniques, such as needle blunting and incremental product waste from repeated needle changes. These factors can influence both patient comfort and economic considerations for practitioners. Multiple‐needle injectors may be advantageous where highly uniform micro‐deposition is desired, although their small aliquots limit real‐time ultrasound confirmation. Collectively, the flexibility to choose among these modalities—without compromising efficacy—supports a more individualized and patient‐centered approach to CPM‐HA20G treatments.

### 4.2 Limitations and Future Directions

This study has limitations, including a relatively small sample size, single‐center design, and a short follow‐up interval that precludes assessment of long‐term persistence. Ultrasound imaging could not capture the micro‐aliquots delivered by the injector device, and the split‐face comparison was a feasibility exercise involving one patient, not a statistically powered evaluation.

Future research should expand the sample size, extend follow‐up, and incorporate more granular mechanistic assessments. AI‐enhanced ultrasound technology represents a promising frontier, with the potential to automatically identify optimal planes, quantify product placement, and assist novice injectors in real time. Additionally, histologic correlation studies could provide objective insight into collagen stimulation, extracellular matrix changes, and comparative tissue responses across modalities. Such work would deepen understanding of the biological impact of CPM‐HA20G and further refine best‐practice recommendations for personalized skin‐quality treatments.

## 5 Conclusion

In summary, this case series demonstrates that CPM‐HA20G can be safely and effectively administered using serial puncture, blunt cannula, or multiple injector, with comparable improvements in hydration, elasticity, and overall skin quality. Ultrasound confirmed consistent subdermal placement across both cannula and serial puncture techniques. No serious AEs were reported. Blunt‐cannula delivery was associated with significantly reduced pain, suggesting potential benefits for patient comfort and adherence. These findings support the flexibility of technique selection in clinical practice, allowing injectors to tailor treatment according to patient needs and preferences without compromising safety or efficacy. Future multicenter trials with objective biophysical measurements and longer follow‐up are warranted to confirm these preliminary findings.

## Funding

This work was supported by Merz Pharmaceuticals.

## Disclosure

The comparative study was sponsored by Merz APAC, which provided financial support for the conduct of the research. The split‐face study was not sponsored. Kim Booysen and Frank Lin have served as speakers for Merz Aesthetics APAC and EMEA.

## Ethics Statement

The authors obtained the informed consents from all of the patients. The study participants gave consent to publish. All patients provided informed consent to publish their case details and any accompanying images.

## Conflicts of Interest

The authors declare no conflicts of interest.

## Data Availability

The data that support the findings of this study are available from the corresponding author upon reasonable request.
